# Finite Time Thermodynamic Modeling and Performance Analysis of High-Temperature Proton Exchange Membrane Fuel Cells

**DOI:** 10.3390/ijms23169157

**Published:** 2022-08-15

**Authors:** Dongxu Li, Zheshu Ma, Wei Shao, Yanju Li, Xinjia Guo

**Affiliations:** College of Automobile and Traffic Engineering, Nanjing Forestry University, Nanjing 210037, China

**Keywords:** HT-PEMFC, finite time thermodynamic, ecological coefficient of performance, exergetic performance efficient

## Abstract

In order to improve the output performance of high-temperature proton exchange membrane fuel cells (HT-PEMFC), a finite time thermodynamic (FTT) model for HT-PEMFC was established. Several finite time thermodynamic indexes including power density, thermodynamic efficiency, exergy efficiency, exergetic performance efficient (EPC), entropy production rate and ecological coefficient of performance (ECOP) were derived. The energetic performance, exergetic performance and ecological performance of the HT-PEMFC were analyzed under different parameters. Results showed that operating temperature, doping level and thickness of membrane had a significant effect on the performance of HT-PEMFC and the power density increased by 58%, 31.1% and 44.9%, respectively. When the doping level reached 8, the output performance of HT-PEMFC wa optimal. The operating pressure and relative humidity had little influence on the HT-PEMFC and the power density increased by 8.7%% and 17.6%, respectively.

## 1. Introduction

As an extension of classical thermodynamics, finite time thermodynamics simulates real systems in a more realistic way, paying more attention to the thermodynamic process of finite size parts in finite time. The basic view of FTT is to seek performance bounds for thermodynamic processes with the goal of reducing the irreversibility of the thermal system under finite time or finite size constraints [1]. The main research contents of finite time thermodynamics include actual performance limits and new evaluation indexes of thermodynamic systems [2,3,4], optimal path and optimal time or size of the thermodynamic process [5], generalized thermodynamic potential or finite time availability [6].

In recent years, the study of finite time thermodynamics has expanded from theoretical cycles to the actual cycles of various systems, including power plants [7,8], refrigerating machines [9,10], heat pumps [11], fuel cells [12,13,14,15,16,17] and other conventional thermal equipment. Compared with the previous studies on HT-PEMFC [12,13,14,15,16,17], this paper not only studies the EPC that takes into account the output power and exergy destruction rate, but also proposes the ECOP that considers both output power and power dissipation, which evaluates the output performance of HT-PEMFC from different perspectives and provides more directions for future performance improvement and optimization. Chen et al. [18] applied finite time thermodynamics to a solar thermal power generation system and found that the energy efficiency of the system increased with the increase in solar heat allocation ratio, and solar irradiance and solar heat allocation ratio both had positive effects on energy and exergy performance. Chen et al. [19] established the thermodynamic model of an irreversible Breton cogeneration device coupled with thermostatic heat source and deduced the dimensionless profit rate and exergetic performance. Results found that the optimal dimensionless profit rate and exergy efficiency had two different internal cooling pressure ratios. Li et al. [20] applied finite time thermodynamics to a solar thermal power generation system and studied the influence of melting temperature of heat storage materials and number of heat storage units on system performance. Compared with the single phase–change material (PCM), using two PCM can increase the overall efficiency by 19–53.8%. For different materials, overall exergy efficiency decreased as the melting temperature of PCM1 increased. As the melting temperature of PCM2 increased, overall exergy efficiency increased. At the same time, compared with the traditional evaluation indexes, the evaluation indexes of ecological factors have many advantages. Yasunaga et al. [3] studied the finite time thermodynamic performance of the organic Rankine cycle and found that efficiency improvement considering finite time or size can more accurately evaluate the energy conversion mechanism of the system. The standardized thermal efficiency of energy conversion, as a new performance index, is effective in the evaluation of energy conversion system. Li et al. [7] optimized the solar-powered Stirling machine model by using finite time thermodynamics, which took finite rate heat transfer, regenerative heat loss and finite regeneration process time into account, and obtained the optimal absorption temperature of about 1100 K and concentration ratio of about 1300 K.

Finite time thermodynamics is a powerful tool for analyzing and optimizing the performance of various thermodynamic cycles and devices [6]. The FTT approach is mainly to analyze the irreversible processes of fuel cells to build different thermodynamic evaluation index models for performance analysis and optimization. In recent years, several authors have used finite-time thermodynamics to analyze and optimize the performance of fuel cells. Liu et al. [21] developed the power system of a proton exchange membrane fuel cell (PEMFC) and established models of entropy production rate, exergy efficiency and ecological function. The study found that the fuel cell stack and heat exchanger were the components that caused the greatest exergetic loss. In the region of low current density, the ecological performance and thermodynamic efficiency of the system were high, but the output power was low. Ye et al. [22] analyzed the influences of different operating parameters on exergetic performance of HT-PEMFC, and the results showed that higher operating temperature was beneficial to improving the exergy efficiency of the system, while operating pressure and relative humidity had less influence. Li et al. [12] analyzed the ecological performance of PEMFC and established models of the ecological objective function and ecological coefficient of performance. The results showed that the water content of the membrane and the operating temperature had a positive effect on the ecological performance of PEMFCs. Guo et al. [23] obtained the optimal operation region of the HT-PEMFC according to the maximum power density criterion but did not optimize and comprehensively determine the parameters of the HT-PEMFC for different performance indicators. Therefore, it is necessary to conduct multi-objective optimization of HT-PEMFCs to determine the optimal operation parameters and design parameters. Akkaya et al. [24] defined the exergetic performance coefficient (EPC) to analyze the performance of solid oxide fuel cells (SOFC). EPC is a thermal–ecological index combining energetic and exergetic performance, which can better analyze the thermodynamic process and cycle.

Due to the merits of green cleaning, high-efficient energy conversion and fast start-up, PEMFC is considered as a promising alternative to the traditional internal combustion engines used in vehicles [25,26,27,28,29,30,31]. However, HT-PEMFC, which has been widely studied in recent years, has more advantages in CO tolerance, water and thermal management [32]. If the HT-PEMFC system is used in vehicles, the problems including performance, stability and durability will impede large-scale commercialization. Liu et al. [33] applied an 80 kW fuel cell system for vehicles and found that higher operating pressure would affect the system efficiency. Li et al. [34] established a HT-PEMFC model of vehicle application in ADVISOR and the results showed that increasing the operating temperature can significantly improve the performance of HT-PEMFCs. Bayat et al. [35] pointed out that increasing the relative humidity contributes to an increase of 8.02% and 8.03% in energy and exergy efficiency. Yang et al. found that increasing the phosphoric acid content in the membrane would improve the proton conductivity. However, if the phosphoric acid content was too high, the mechanical strength of the membranes would be decreased. Therefore, exploring the effect of operating and designing parameters on HT-PEMFCs is an important research area, which will make a great contribution in HT-PEMFCs applied in the vehicle.

At present, there have been numerous experimental and theoretical studies on the HT-PEMFC single cell. Zhou et al. [36] found that when the operating temperature of HT-PEMFC is below 140 °C, the performance could be improved by reducing the water content of hydrogen. When the operating temperature is above 140 °C, the performance is improved with the increase in water content. Sousa et al. [37] established a two-dimensional isothermal model of HT-PEMFC with phosphoric acid doped polybenzimidazole (PBI) membrane and verified the accuracy of the model through experimental data. The study found that improving the phosphoric acid doping level can significantly improve the performance of HT-PEMFC. Guo et al. [23] studied the effects of operating pressure, operating temperature, doping level and relative humidity on the performance of HT-PEMFC. Lu et al. [38] studied the influence of operating pressure and operating temperature on the HT-PEMFC power generation system. Guo et al. [39] established a waste heat recovery system based on HT-PEMFC and studied the influence of current density and operating temperature on system performance.

Many previous studies have used density functional theory (DFT) to predict many of the energetic and dynamic effects in proton fuel cells to provide insight into these materials [40,41]. However, the focus of this paper is to analyze the thermodynamic performance of the fuel cell when using polybenzimidazole (PBI) membranes under different operating conditions. This paper develops a fuel cell model based on FTT not only to predict the performance of ion membrane in fuel cells but also to explore the thermodynamic performance under different parameters to provide a direction for optimization. To improve the performance of HT-PEMFCs, this paper develops the mathematical models of power density, thermodynamic efficiency, exergy efficiency, entropy production rate, ecological coefficient of performance and exergetic performance coefficient of HT-PEMFCs. In addition, operating temperature, operating pressure, doping level, relative humidity and thickness of membrane are used as significant parameters to analyze the performance of HT-PEMFC.

## 2. Results and Discussion

### 2.1. Working Principle of HT-PEMFC

**Figure 1:**
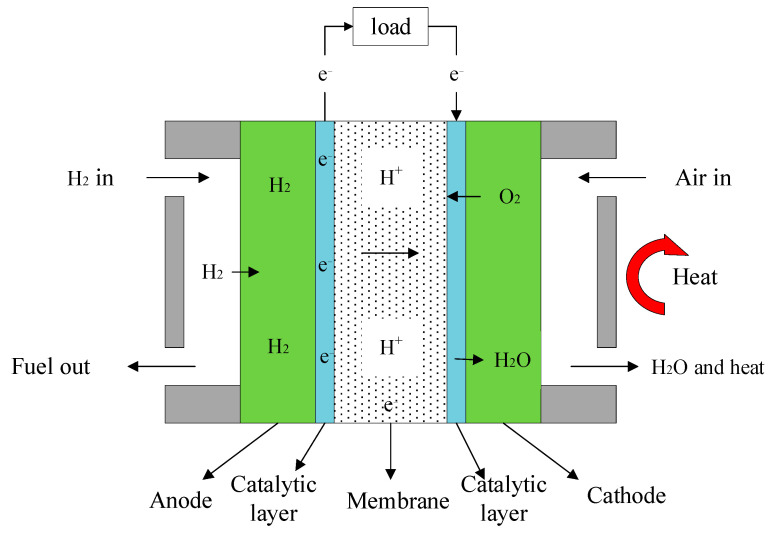
Working principle of HT-PEMFC.

As shown in Figure 1, HT-PEMFC consists of a cathode, anode and electrolyte. When HT-PEMFC works, hydrogen enters the anode, oxygen or air enters the cathode. Hydrogen reacts at the anode to become protons and electrons, then hydrogen ions pass through the proton exchange membrane to the cathode, and electrons pass through the external load to the cathode. Oxygen reacts with protons and electrons to form H_2_O after the reduction reaction at the cathode.

The electrochemical reaction equations of the HT-PEMFC are:

(1) $$\mathrm{Anode}\;\mathrm{reaction}:H_{2}\to 2H^{+}+2e^{-}$$

(2) $$\mathrm{Cathode}\;\mathrm{reaction}:2H^{+}+\frac{1}{2}O_{2}+2e^{-}\to H_{2}O+\mathrm{heat}$$

(3) $$\mathrm{Total}\;\mathrm{reaction}:H_{2}+\frac{1}{2}O_{2}\to H_{2}O+\mathrm{heat}+\mathrm{electricity}$$

Compared with the LT-PEMFC, phosphoric acid replaces water in HT-PEMFC. The mass transfer principle in anode, cathode and membrane can be expressed as:

(4) $$\mathrm{Anode}:H_{2}{\mathrm{PO}}_{4}^{-}+H^{+}=H_{3}{\mathrm{PO}}_{4}$$

(5) $$\mathrm{Membrane}:H_{3}{\mathrm{PO}}_{4}+\mathrm{PBI}=H_{2}{\mathrm{PO}}_{4}^{-}+\mathrm{PBI}\cdot H^{+}$$

(6) $$\mathrm{Cathode}:\;\mathrm{PBI}\cdot H^{+}=\mathrm{PBI}+H^{+}$$

In the HT-PEMFC system, the electrochemical reaction potential depends on the Gibbs free energy of fuel. The ideal standard potential of HT-PEMFC at 298 K and 1 atm can be expressed as:

(7) $$E_{r}^{0}=-\frac{\Delta g^{0}}{nF}$$

where $\Delta g^{0}$ is molar Gibbs free energy change of the electrochemical reaction at 298 K and 1 atm. The ideal standard potential of HT-PEMFC is 1.185 V. For HT-PEMFCs, reversible potential can be given as follows:

(8) $$E_{\mathrm{rev}}=E_{r}^{0}+\frac{\Delta S}{nF}(T-298.15)+\frac{\mathrm{RT}}{\mathrm{nF}}\ln(\frac{p_{H_{2}}p_{O_{2}}^{0.5}}{p_{H_{2}O}})$$

where T is the operating temperature of HT-PEMFC; $p_{H_{2}}$, $p_{O}$ and $p_{H_{2}O}$ are the partial pressure of $H_{2}$, $O_{2}$ and $H_{2}O$, respectively.

### 2.2. Voltage Model of HT-PEMFC

For HT-PEMFCs, the actual output voltage is less than the reversible voltage resulting from kinds of overpotential including activation overpotential ($E_{act}$), ohmic overpotential ($E_{ohm}$) and concentration overpotential ($E_{con}$). The actual output voltage (U) can be calculated by:

(9) $$U=E_{rev}-E_{con}-E_{act}-E_{ohm}=E_{rev}-\left(1+\frac{1}{\alpha }\right)\frac{RT}{nF}\ln(\frac{j_{L}}{j_{L}-j})-\frac{RT}{n\alpha F}\ln(\frac{j+j_{leak}}{j_{0}})-j\frac{t_{mem}}{\sigma_{mem}}$$

where α is the charge transfer coefficient; $j_{L}$ is the limiting current density; $j_{leak}$ is the leakage current density; $j_{0}$ is the exchange current density; $t_{mem}$ is the thickness of the membrane, $\sigma_{mem}$ is the proton conductivity.

According to the experimental study, Santerelli et al. [42] found that the leakage current density of LT-PEMFC increased with the rise of the operating temperature. When the operating temperature was 50–80 °C, the leakage current density reached 60–120 A/m^2^. Guo et al. [23] took the leakage current density as a constant value of 50 A/m^2^ when studying the performance of HT-PEMFC. Shaker [43] obtained the leakage current density when PEMFC operated at a certain temperature through an experimental study. Based on previous studies on the leakage current density of HT-PEMFC, the leakage current density model adopted in this paper is shown as follows [43]:

(10) $$\ln j_{leak}=\left(-2342.9(\frac{1}{T})+9.0877\right)$$

### 2.3. Power Density and Thermal Efficiency

The output power density of HT-PEMFC is shown as follows:

(11) $$P=jU=(E_{rev}-E_{act}-E_{ohm}-E_{con})j$$

For an energy conversion device, the thermal efficiency is the output energy divided by the total input energy. The total released energy is expressed as:

(12) $$\Delta \dot{H}=-\frac{jA\Delta h}{nF}$$

where Δh is the change of molar enthalpy.

The thermal efficiency of the HT-PEMFC can be represented by:

(13) $$\eta =\frac{P}{-\Delta \dot{H}}=\frac{(E_{rev}-E_{act}-E_{ohm}-E_{con})j}{-\Delta \dot{H}}=\frac{(E_{rev}-E_{act}-E_{ohm}-E_{con})nF}{A\Delta h}$$

According to the first law of thermodynamics, the remaining part of the thermal rate (${\dot{Q}}_{H}$) of HT-PEMFC can be represented by:

(14) $${\dot{Q}}_{H}=-\Delta \dot{H}-P-{\dot{Q}}_{L}=\frac{jA\Delta h}{\mathrm{nF}}-P-K_{L}A_{L}(T-T_{0})=\frac{A}{\mathrm{nF}}[-(1-\eta )j\Delta h-\frac{nFK_{L}A_{L}}{A}(T-T_{0})]$$

where ${\dot{Q}}_{L}$ is heat-leakage rate from HT-PEMFC to the environment; $K_{L}$ is the heat leak coefficient; $A_{L}$ is the corresponding area.

### 2.4. Exergy Efficiency and Exergetic Performance Coefficient

During the operation of HT-PEMFCs, energy loss and decrease in output performance results from irreversibility of heat loss, friction between gas and channel, leakage current and polarization. To completely evaluate the thermodynamic performance of HT-PEMFCs, the exergy analysis is necessary to be applied according to the second law of thermodynamics. Exergetic performance analysis of HT-PEMFCs provides a criterion for evaluating the quality of released energy. Exergy is defined as the maximum available work or the minimum available work loss in a reversible process. For any thermodynamic process, exergy transfer can be regarded as the sum of specific physical exergy, chemical exergy, kinetic exergy and potential exergy. For HT-PEMFCs, only physical exergy ($\varepsilon_{phy}$) and chemical exergy ($\varepsilon_{chem}$) are considered.

(15) $$ex=ex_{chem}+ex_{phy}$$

The physical exergy and the chemical exergy of a substance can be expressed as follows:

(16) $$ex_{chem}=\sum x_{i}ex_{chem,i}^{0}+RT_{0}\sum x_{i}\ln x_{i}$$

(17) $$ex_{phy}=\left(h-h_{0}\right)-298.15\left(s-s_{0}\right)+\left(\frac{v^{2}-v_{0}^{2}}{2}\right)+g\left(z-z_{0}\right)$$

where $x_{i}$ is a molar fraction; $ex_{chem,i}^{0}$ is standard chemical exergy; i is $H_{2}$, $O_{2}$ and $H_{2}O$; v, g and z are velocity, gravity and elevation, respectively. In this paper, only gravity is considered, therefore the expression is:

(18) $$ex_{phy}=\left(h-h_{0}\right)-298.15\left(s-s_{0}\right)$$

where $\left(h-h_{0}\right)$ and $\left(s-s_{0}\right)$ are the change of molar enthalpy and entropy, respectively.

In HT-PEMFCs, the total input and output exergy rates are shown as follows:

(19) $${\dot{ex}}_{in}=\frac{jA}{nF}\left(ex_{H_{2}}+0.5ex_{O_{2}}\right)$$

(20) $${\dot{ex}}_{out}=\frac{jA}{nF}ex_{H_{2}O}$$

Exergy efficiency of HT-PEMFC reflects the effective utilization degree of exergy, and its expression is as follows:

(21) $$\varphi =\frac{P}{{\dot{ex}}_{in}}$$

Energy loss is inevitable in all irreversible processes, including heat transfer, fuel mixing, chemical friction and polarization phenomena. The irreversible loss is usually described by the exergy destruction rate, which can be used to measure the degree of irreversible loss. The greater the exergy destruction, the greater the irreversible loss of the HT-PEMFC thermodynamic process, and the less effective utilization of exergy destruction. Exergy destruction rate can be shown as:

(22) $$ExD={\dot{ex}}_{in}-{\dot{ex}}_{out}-P+{\dot{Q}}_{H}\left(1-\frac{T_{0}}{T}\right)$$

In order to evaluate the HT-PEMFC better, a thermal–ecological index, which is the exergetic performance coefficient, is used to analyze the HT-PEMFC. It combines thermodynamic and exergetic performance, defined as the ratio of output power to exergy destruction rate. EPC can be expressed as:

(23) $$EPC=\frac{P}{ExD}=\frac{jUA}{{\dot{ex}}_{in}-{\dot{ex}}_{out}-P+{\dot{Q}}_{H}\left(1-\frac{T_{0}}{T}\right)}$$

### 2.5. Entropy Production Rate and Ecological Coefficient of Performance

According to finite time thermodynamics, not only the energetic and exergetic indexes can be used to evaluate the HT-PEMFC, but also the ecological standards. Angulo [44] proposed the ecological objective function (E) as the optimized objective of the Carnot engine. This objective function considers the output power and the power dissipation, so that the objective conforms to the long-term ecological principle [44]. On this basis, ecological coefficient of performance (ECOP) proposed by Ust [45,46] is an important ecological index, which is the ratio of output power to power dissipation. Compared with the ecological objective function, ECOP improve the relationship between output power and power dissipation. It can be calculated as:

(24) $$ECOP=\frac{PA}{T_{0}\dot{\delta }}$$

where $\dot{\delta }$ is the entropy production rate which means the increasing rate of entropy. $\dot{\delta }$ can be expressed as follows:

(25) $$\dot{\delta }=\frac{-\Delta \dot{H}-P}{T}=\frac{\frac{jA\Delta h}{nF}-\left[E_{rev}-\left(1+\frac{1}{\alpha }\right)\frac{RT}{nF}\ln(\frac{j_{L}}{j_{L}-j}\right)-\frac{RT}{n\alpha F}\ln(\frac{j+j_{leak}}{j_{0}})-j\frac{t_{mem}}{\sigma_{mem}}]j}{T}\;$$

Due to the numerical differences among the indicators, the dimensionless method is used to better compare the effects of different parameters on the indicators. The dimensionless function of power density, thermal efficiency, entropy production rate, exergetic performance coefficient, exergy efficiency and ecological coefficient of performance can be expressed as:

(26) $$\bar{P}=\frac{P}{P_{max}}$$

(27) $$\bar{\eta }=\frac{\eta }{\eta_{max}}$$

(28) $$\bar{\dot{\delta }}=\frac{\dot{\delta }}{{\dot{\delta }}_{max}}$$

(29) $$\bar{EPC}=\frac{EPC}{EPC_{max}}$$

(30) $$\bar{\varphi }=\frac{\varphi }{\varphi_{max}}$$

(31) $$\bar{ECOP}=\frac{ECOP}{ECOP_{max}}$$

In order to better compare the impact of different parameters on the performance index of HT-PEMFC, the dimensionless method is also adopted in different parameters, including operating temperature (*T* = 413 K, 443 K, 473 K), operating pressure (*p* = 1 atm, 2 atm, 3 atm), relative humidity (*RH* = 0, 3.8%, 7.6%), the phosphoric acid doping level (*DL* = 4, 6, 8, 10) and membrane thickness ($t_{mem}\;$= 0.002 cm, 0.006 cm, 0.010 cm). The phosphoric doping level, depending on the phosphoric acid concentration, doping temperature and soaking time, is defined as the number of phosphoric acid molecules per polybenzimidazole [47]. The dimensionless expression of operating temperature is as follows:

(32) $${\bar{T}}_{1}=\frac{T_{1}}{T_{max}}=\frac{413\;K}{473\;K}$$

(33) $${\bar{T}}_{2}=\frac{T_{2}}{T_{max}}=\frac{443\;K}{473\;K}$$

(34) $${\bar{T}}_{3}=\frac{T_{3}}{T_{max}}=\frac{473\;K}{473\;K}$$

The dimensionless expression of operating pressure, relative humidity, doping level and membrane thickness can be derived similarly to the dimensionless operating temperature.

## 3. Materials and Methods

All analyses are performed based on the following assumptions:

(1) The HT-PEMFC is working in a steady state;
(2) The operating temperature and the operating pressure are constant at a fixed time;
(3) All gases within the HT-PEMFC are assumed to be ideal gas;
(4) Anode outlet temperature is equal to the operating temperature;
(5) The effect of CO poisoning is negligible.

Based on the present study on HT-PEMFC single cell [36,37,38,39], the input parameters of HT-PEMFC based on finite-time thermodynamics in this paper are shown in Table 1.

**Figure 2:**
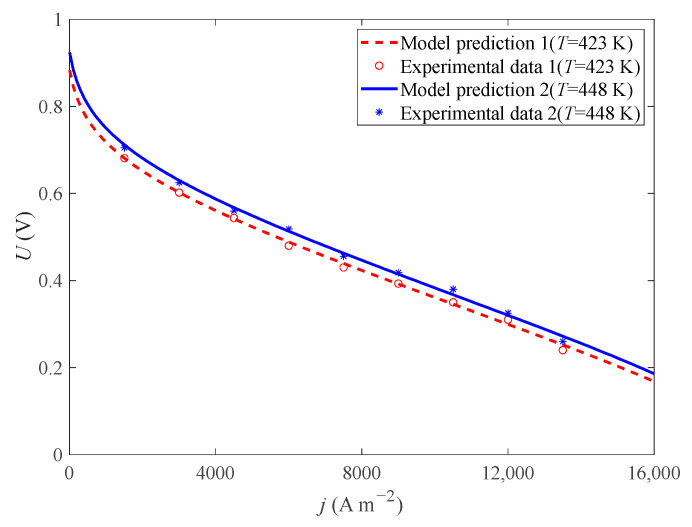
Comparison of experimental and simulation results under different T.

According to the test conditions in reference [37] and the model parameters in Table 1, this paper compares the experimental voltage and model voltage under different operating temperatures (*T* = 423 K and *T* = 448 K) (*p* = 1 atm, *DL* = 5.6, *RH* = 0.38%). As shown in Figure 2, the result shows that the model voltage is basically consistent with the experimental voltage.

### 3.1. Operating Temperature

The phosphoric acid-doped polybenzimidazole (PBI) membrane used in HT-PEMFCs is a commonly used high-temperature membrane [48,49,50], which can work for a long time in a high temperature (413 K–473 K) and has stable chemical properties and good hydrogen ion passage. Compared with LT-PEMFC, HT-PEMFC has higher output efficiency, and is more resistant to CO poisoning.

**Figure 3:**
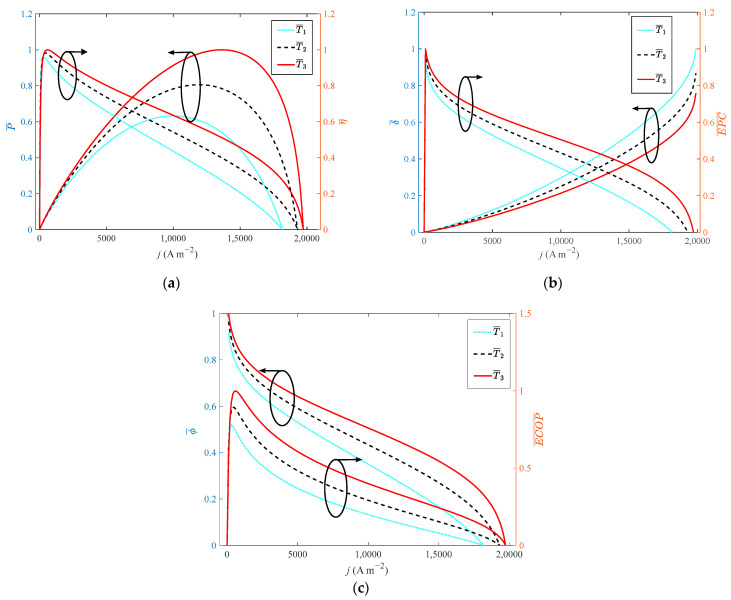
Different indexes vary with current density at different operating temperatures: (**a**) *P* and *η*; (**b**) *δ* and *EPC*; (**c**) *φ* and *ECOP*.

It can be seen from Figure 3 that increasing the operating temperature of HT-PEMFC has a significant impact on its output performance. When the operating temperature rises from 413 K to 473 K with a step width of 30 K, the growth rate of the operating temperature is 14.52%. The dimensionless of *P*, *η*, *EPC*, φ and *ECOP* increase by 58%, 4%, 9%, 9% and 28%, respectively. The dimensionless of $\dot{\delta }$ decreases by 24%. The main reasons are as follows: increasing the operating temperature, the activity of the cathode and anode catalysts is enhanced, and the electrochemical reaction rate of hydrogen and oxygen is accelerated. The diffusion coefficient of the reaction gas is increased, and the mass transfer of gas in the electrode is improved.

### 3.2. Operating Pressure

**Figure 4:**
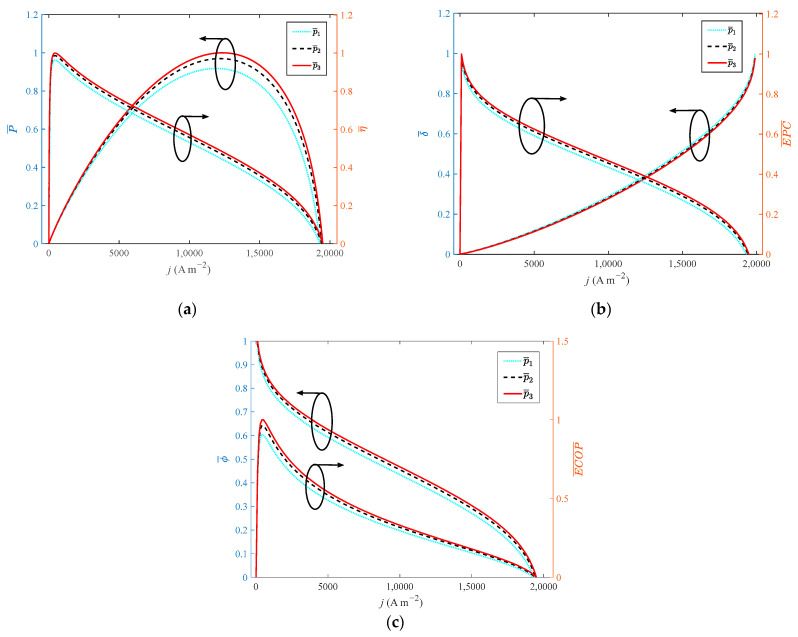
Different indexes vary with current density at different operating pressures: (**a**) *P* and *η*; (**b**) *δ* and *EPC*; (**c**) *φ* and *ECOP*.

Figure 4 shows the variation of different indexes with current density at different operating pressures. From the perspective of electrochemical and thermodynamics, the increase in operating pressure can enhance the diffusion rate of the gas, and optimize the reaction rate of the reaction gas. When the operating pressure increases from 1 atm to 3 atm with a step width of 1 atm, the growth rate of operating pressure is 200%. However, the dimensionless of P, η, EPC, φ and ECOP only increase by 8.7%, 3.7%, 3.4%, 3.4% and 10.3%, respectively. The dimensionless of $\dot{\delta }$ reduces by 2%. Therefore, operating pressure has little effect on the performance of HT-PEMFC. In actual application, the operating pressure is usually set as 1 atm to simplify the HT-PEMFC system.

### 3.3. Relative Humidity

**Figure 5:**
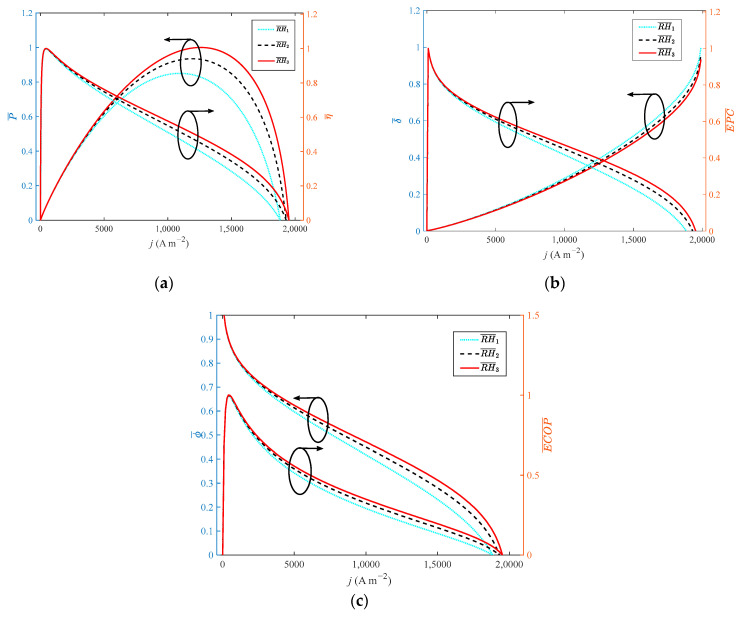
Different indexes vary with current density at different relative humidity: (**a**) *P* and *η*; (**b**) *δ* and *EPC*; (**c**) *φ* and *ECOP*.

Figure 5 presents the variation of different indexes with current density at different relative humidity.Compared with the LT-PEMFC, the high-temperature membrane of HT-PEMFC can still work without humidification, but the proton conductivity decreases. The relative humidity is an important factor affecting proton conductivity, and an increase in relative humidity is beneficial to the improvement of proton conductivity. When the relative humidity increases from 0 to 7.6% with a step width of 3.8%, the growth rate of relative humidity is 100%. From the numerical perspective, relative humidity increases dramatically, but the dimensionless of *P*, *η*, *EPC*, *φ* and *ECOP* only grow by 17.6%, 0.2%, 0.05%, 0.05% and 0.6%, respectively. The dimensionless of $\dot{\delta }$ reduces by 6.9%.

### 3.4. Doping Level

**Figure 6:**
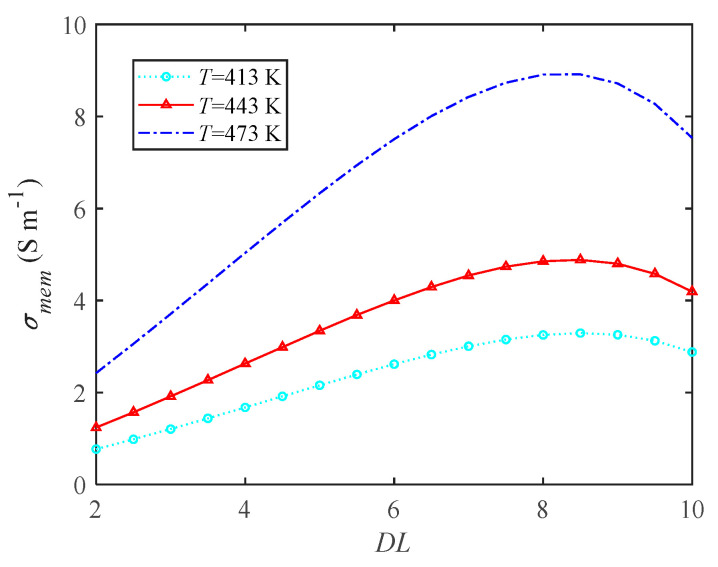
The influence of doping level on proton conductivity.

The high-temperature membrane of HT-PEMFC is a PBI membrane. The doping level (*DL*) of a PBI membrane refers to the ratio of the mass difference between after acidification and before acidification to the mass before acidification. The proton conductivity $\sigma_{mem}$ is a function of the doping level, and the specific relationship is shown in Figure 6. With the increase in phosphoric acid doping level, the proton conductivity first increases and then decreases. When the DL is 8, the proton conductivity reaches the maximum value, and the ohmic overpotential is the smallest at this time. Moreover, increasing the operating temperature of HT-PEMFC can significantly improve the proton conductivity, therefore the operating temperature has a great influence on the performance.

**Figure 7:**
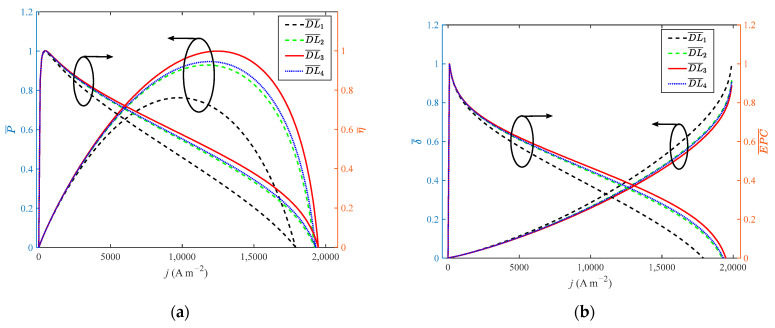

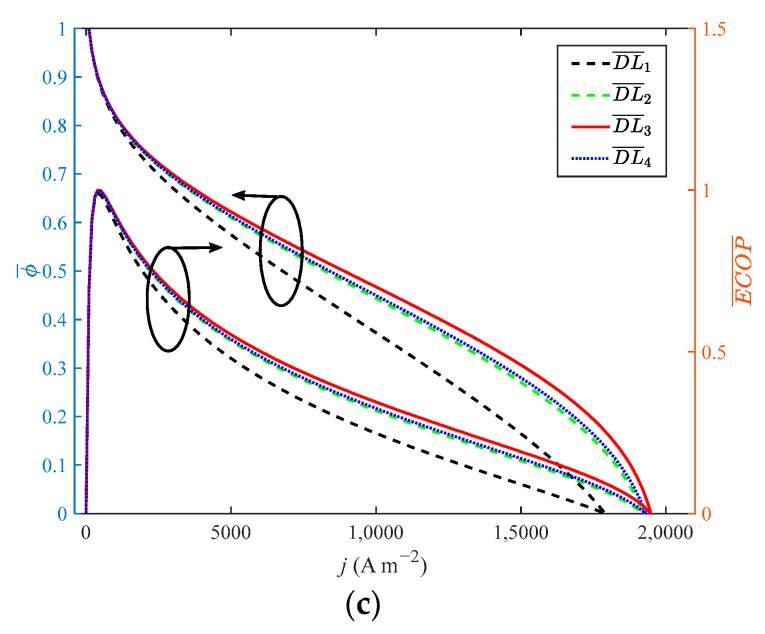
Different indexes vary with current density at different doping level: (**a**) *P* and *η*; (**b**) *δ* and *EPC*; (**c**) *φ* and *ECOP*.

As shown in Figure 7, when the doping level increases from 4 to 10 with a step width of 2, the growth rate of the doping level is 150%. When DL reaches 8, the performance of HT-PEMFC is optimal. When the growth rate of the doping level is 100%, the dimensionless of *P*, *η*, *EPC*, *φ* and *ECOP* only grow by 31.1%, 0.4%, 0.09%, 1.1% and 1%, respectively. The dimensionless of $\dot{\delta }$ reduces by 11%. Compared with Figure 4 and Figure 5, the growth rate of power density is more obvious. Guo et al. [23] found that a higher doping level is beneficial to the performance of HT-PEMFCs. However, if the doping level is too high, phosphoric acid will be easier to leave out of the membrane. Therefore, 8 is considered to be the optimal value for *DL*.

### 3.5. Membrane Thickness

Membrane thickness ($t_{mem}$) is one of the main factors of waste heat generation. With the rise of the thickness of the high-temperature membrane, the path length of ion between anode and cathode is increased, resulting in the increase in ohmic overpotential of HT-PEMFC. Therefore, the thinner film should be selected as the membrane. However, if the membrane is too thin, HT-PEMFC will have fuel penetration, short circuit, membrane rupture and other problems. Therefore, it is necessary to study the influence of $t_{mem}$ on the output performance of HT-PEMFC.

**Figure 8:**
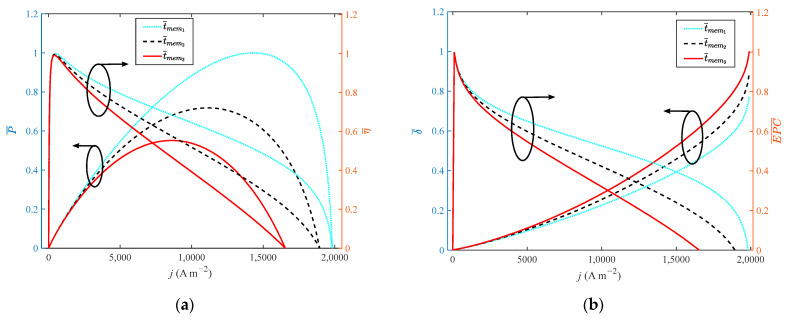

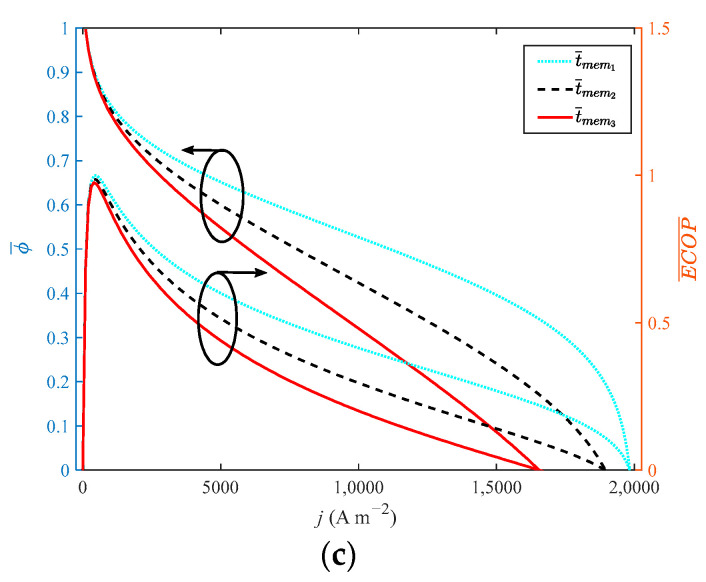
Different indexes vary with current density at different membrane thicknesses: (**a**) *P* and *η*; (**b**) *δ* and *EPC*; (**c**) *φ* and *ECOP*.

It can be seen from Figure 8 that when the thickness of the membrane increases from 0.002 cm to 0.01 cm with a step width of 0.004 cm, the growth rate of the doping level is 400%. As the performance of HT-PEMFC is reduced by increasing film thickness, the dimensionless of P, η, *EPC*, *φ* and ECOP only grow by 31.1%, 0.4%, 0.09%, 1.1% and 1%, respectively. The dimensionless of $\dot{\delta }$ reduces by 11%. It is necessary to select a proper value for the thickness of a high-temperature membrane.

### 3.6. Engineering Application of HT-PEMFC in Vehicle

When HT-PEMFCs are used in fuel cell vehicles (FCVs), the external characteristic curve of the fuel cell system is of great significance to study the dynamic performance of FCVs. According to the related reference of FCVs, the external characteristic curve consisting of power density and thermal efficiency is an important indicator for evaluating the dynamic performance of FCVs.

**Figure 9:**
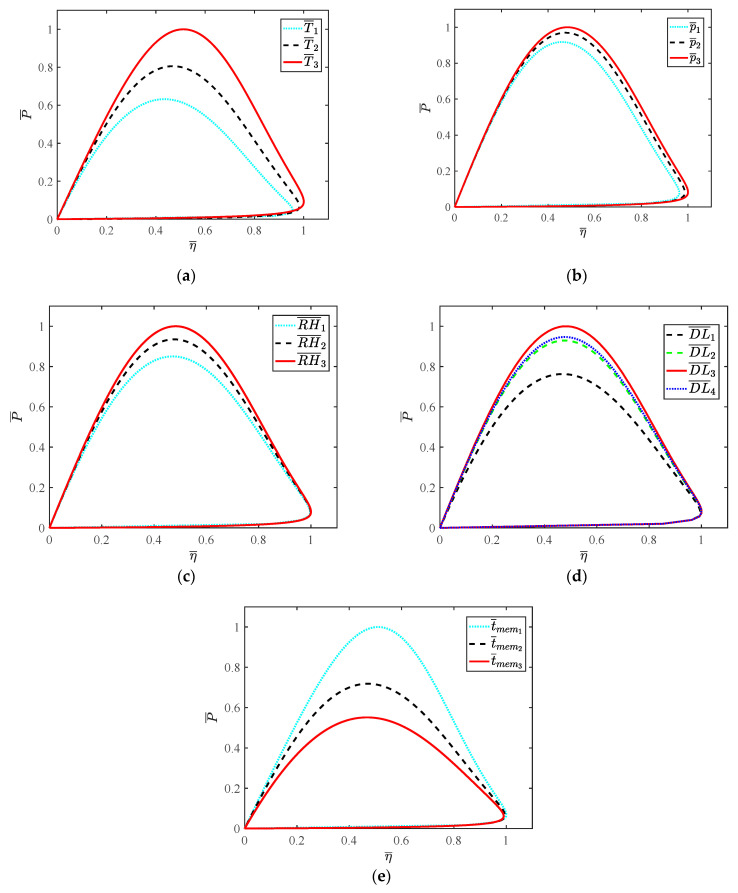
The relationship between power density and thermal efficiency: (**a**) $\bar{T}$; (**b**) $\bar{p}$; (**c**) $\bar{RH}$; (**d**) $\bar{DL}$; (**e**) ${\bar{t}}_{\mathrm{mem}}$.

As shown in Figure 9, the relationship between power density and thermal efficiency of HT-PEMFC is obtained. The effects of different parameters on the performance of HT-PEMFC can be significantly compared by the dimensionless method. Obviously, operating temperature, doping level and membrane thickness can greatly improve the performance of HT-PEMFCs. In view of operating temperature, it is necessary to design a reasonable thermal management system and corresponding control strategy. With the increase in operating temperature, the polarization loss decreases and the output voltage increases. In addition, with the increase in the load current, the electrochemical reaction in HT-PEMFCs is strengthened and the performance difference is increased. However, doping level and membrane thickness are the designing parameters of HT-PEMFC, they cannot be easily modified after production. Therefore, the influence of doping level and membrane thickness in HT-PEMFC analyzed in this paper can provide a theoretical basis and reference for future production. Although operating pressure and relative humidity have little effect on the performance of HT-PEMFCs, it is essential to take corresponding measures to control these two parameters. For the intake gas, the air compressor is usually used to increase the pressure, so as to improve the performance of the cathode side of the HT-PEMFCs. Additionally, the unreacted hydrogen can be recycled by installing a hydrogen recycling pump at the outlet of the anode. The humidifier is added to the HT-PEMFC system to increase the relative humidity. With the increase in relative humidity, the impedance of the proton exchange membrane is reduced and the electrochemical reaction of PEMFC is accelerated. The above analysis can provide guidance for the design of FCVs.

## 4. Conclusions

In order to evaluate the thermodynamic performance of the HT-PEMFC, finite time thermodynamics was used to establish the HT-PEMFC model which considers three kinds of overpotential and leakage current density. The expressions of power density, thermal efficiency, exergy efficiency, exergetic performance coefficient (EPC), entropy production rate and ecological coefficient of performance (ECOP) were deduced. By analyzing the influence of main parameters on HT-PEMFC, it was found that operating temperature, doping level and membrane thickness have a great influence on HT-PEMFC. With the increase in operating temperature, doping level and decrease in membrane thickness, the performance of HT-PEMFC was better. In addition, when the doping level reached 8, the performance of HT-PEMFC was optimal. However, the effect of operating pressure and relative humidity on HT-PEMFC was insignificant. The effect of different parameters on HT-PEMFC can provide guidance for the design of HT-PEMFC. For example, it is necessary to design reasonable thermal management and control strategy to control the operating temperature. The air compressor, hydrogen recycling pump and humidifier should be installed to increase the operating pressure, recycle the unreacted hydrogen and improve the relative humidity, respectively. In further research, it will be necessary to study the practical engineering application of HT-PEMFC stacks in vehicles according to the FTT.

## Figures and Tables

**Table 1:** Parameters of HT-PEMFC model.

Parameters	Values
Environment temperature, *T*_0_ (K)	298.15
Faraday constant, *F* (C/mol)	96,485
Gas constant, *R* (J mol^−1^ K)	8.314
Charge transfer coefficient, *α*	0.25
Current density, *j* (A m^−2^)	0–20,000
Operating temperature, *T* (K)	413–473
Operating pressure, *p* (atm)	1–3
Relative humidity, *RH* (%)	0–7.6
The phosphoric acid doping level, *DL*	2–10
The thickness of membrane, *t_mem_* (cm)	0.002–0.010
Activation area, *A* (cm^2^)	600

## Data Availability

Not applicable.
